# Non-Canonical WNT5A Signaling Through RYK Contributes to Aggressive Phenotype of the Rheumatoid Fibroblast-Like Synoviocytes

**DOI:** 10.3389/fimmu.2020.555245

**Published:** 2020-10-15

**Authors:** Angela Rodriguez-Trillo, Nerea Mosquera, Carmen Pena, Fatima Rivas-Tobío, Antonio Mera-Varela, Antonio Gonzalez, Carmen Conde

**Affiliations:** ^1^Laboratorio de Reumatología Experimental y Observacional, y Servicio de Reumatología, Instituto de Investigación Sanitaria de Santiago (IDIS), Hospital Clinico Universitario de Santiago de Compostela (CHUS), Servizo Galego de Saude (SERGAS), Santiago de Compostela, Spain; ^2^Servicio de Cirugía Ortopédica y Traumatología, Hospital Virxe da Xunqueira, A Coruña, Spain; ^3^Servicio de Reumatología, Instituto de Investigación Sanitaria de Santiago (IDIS), Hospital Clinico Universitario de Santiago de Compostela (CHUS), Servizo Galego de Saude (SERGAS), Santiago de Compostela, Spain

**Keywords:** rheumatoid arthritis, fibroblast-like synoviocytes, migration, invasion, inflammatory response, WNT5A, RYK, MAPK

## Abstract

We hypothesized that WNT5A could contribute to the enhanced migration and invasiveness of rheumatoid arthritis fibroblast-like synoviocytes (RA FLS), which is one of the incompletely understood aspects of the RA FLS aggressive phenotype. This hypothesis is based on the previous evidence of a WNT5A role in both, RA and cell migration. Migration and invasion of RA FLS were assessed after incubation with recombinant Wnt5a (rWnt5a) or silencing of the endogenous WNT5A expression. The expression of WNT5A, WNT receptors, cytokines, chemokines, and metalloproteinases was quantified with RT-PCR. The WNT pathway was explored with gene silencing, antibody and pharmacological inhibition followed by migration assays and phosphoprotein western blots. Here, we reported that rWnt5a promoted migration and invasion of RA FLS, whereas knockdown of the endogenous WNT5A reduced them. These effects were specific to the RA FLS since they were not observed in FLS from osteoarthritis (OA) patients. Also, rWnt5a induced the expression of IL6, IL8, CCL2, CXCL5, MMP1, MMP3, MMP9, and MMP13 from baseline or potentiating the TNF induction, WNT5A signaling required the RYK receptor and was mediated through the WNT/Ca^2+^ and the ROCK pathway. These pathways involved the RYK and ROCK dependent activation of the p38, ERK, AKT, and GSK3β kinases, but not the activation of JNK. Together these findings indicate that WNT5A contributes to the enhanced migration and invasiveness of RA FLS through RYK and the specific activation of ROCK and downstream kinases.

## Introduction

Rheumatoid arthritis (RA) is an autoimmune disease characterized by chronic inflammation and progressive destruction of the peripheral joints (1, 2), in which the resident fibroblast-like synoviocytes (FLS) are pivotal. These cells contribute to joint inflammation and damage by secreting inflammatory mediators, metalloproteinases, and cathepsins and by invading periarticular cartilage and bone. These actions are manifestations of the transformed-like phenotype the RA FLS show, which is also characterized by over-expression of proto-oncogenes, mutations of several tumor suppressor genes, increased proliferation, resistance to apoptosis and to contact-inhibition (3–5). This aggressive phenotype is maintained over time even in the absence of external inflammatory stimulation (6). Although recent research has been focused on the study of this phenotype (7–10), the mechanisms of many aspects of the RA FLS transformation, as their increased migration and invasiveness, are far from been completely known.

We have hypothesized that WNT5A, a member of the secreted glycoproteins family Wingless/integrase 1, will have a major role because it is known to participate in similar migration or invasion processes in health and disease and to be overexpressed in the RA FLS. In effect, WNT5A is involved in cytoskeleton remodeling, tissue polarization, cell migration and axon guidance in healthy tissues (11–16) and promotes migration and invasion in cancer cells (17–19). These actions are mediated through the non-canonical or β-catenin independent WNT pathways, which are the specific pathways transmitting WNT5A signals. Specifically, WNT5A binds a variety of receptors on the cell surface. They include the FDZ receptors that are shared with the WNT canonical pathway, but also other receptors, ROR1, ROR2, and RYK, which are specific of the non-canonical WNTs and WNT5A does not bind the canonical pathway coreceptors, LRP5/6 (20–24). In addition, the non-canonical pathways do not mediate their signals through the stabilization and nuclear translocation of β-catenin as the canonical pathway does (21). In contrast, the non-canonical signals follow either the WNT/Ca^2+^ or the PCP (planar cell polarity) pathways (13, 14, 20). In the WNT/Ca^2+^ signaling, the Wnt ligand-receptor interaction leads to the release of intracellular calcium, which activates protein kinase C (PKC), calmodulin-dependent protein kinase II (CaMKII), or calcineurin (CaN). The alternative PCP pathway involves the downstream activation of Rho family small GTPases, including Cdc42, Rac, and RhoA. In turn, activated Rac and Cdc42 initiate downstream c-Jun N-terminal kinase (JNK)/activating protein-1 (AP-1) signal transduction, whereas activated RhoA leads to Rho-Kinase (ROCK) activation. In this way, the PCP pathway regulates the cytoskeleton remodeling, tissue polarity, coordinated cell migration and axon guidance, and the WNT/Ca^2+^ pathway regulates cell migration, fate determination, and axon guidance and cooperates with the PCP pathway in controlling tissue polarity (13, 14, 20). Dysregulation of the WNT5A signaling has been implicated in the increased migration and invasiveness of cancer cell lines (17–19) and the metastatic potential of gastric, breast and nasopharyngeal cancers (19, 25, 26).

Apart from the WNT5A effects related to migration and invasion, which have not been studied in RA yet, there are some pieces of evidence indicating the involvement of WNT5A in inflammatory diseases, and specifically in RA. For example, expression of WNT5A is increased in synovial tissue from RA patients, both in synoviocytes and endothelial cells (27), and WNT5A overexpression in normal fibroblast induces the expression of IL6, IL8, and IL15 (28). A result that was complemented by the demonstration that blockade of WNT5A in RA FLS reduces the expression of IL6 and IL15 (29). The stimulation of pro-inflammatory cytokines by WNT5A seems to result from NFκB activation through Wnt/Ca^2+^/protein kinase C pathway in endothelial cells (27) or CaMKII in macrophages (30), or through the PCP pathway also in macrophages (31) and microglia (32). Also, the inducible *Wnt5a* deficiency in mice reduced the severity of arthritis in the K/BxN serum-transfer model, an effect that has been attributed to decreased inflammation and osteoclastogenesis (33). The latter interpretation is supported by the role of WNT5A in inducing osteoclastogenesis by a pathway requiring the presence of ROR2 and leading to the activation of JNK in mice (34). Therefore, accumulating evidence shows the involvement of WNT5A in RA through a variety of mechanisms and pathways but without addressing FLS migration and invasion.

## Material and Methods

### Patients and Cell Culture

FLS were obtained from the synovial tissue of 11 RA patients undergoing synovectomy and 5 patients with osteoarthritis (OA) at the time of total joint replacement. The patients fulfilled the ACR/EULAR criteria for the classification of RA (35, 35) and all patients provided informed written consent. The study was performed according to the recommendations of the Declaration of Helsinki and was approved by the Comité de Ética de Investigación de Santiago-Lugo.

FLS were obtained by digestion of synovial tissue as previously described (36). Adherent cells at 80% to 90% confluence were trypsinized and diluted at a split ratio of 1:3. Only FLS at passages 3 and 8 were used for experiments. Cells were treated when indicated with TNF (10 ng/ml, Sigma Aldrich), recombinant Wnt5a (400 ng/ml, R&D, Biotechne, Minneapolis, USA), anti-tyrosine-protein kinase RYK antibody (1 µg/ml, Abgent, Inc. San Diego, USA), anti-tyrosine-protein kinase ROR2 antibody (4 µg/ml; OriGene), normal rabbit IgG (1 µg/ml; Cell Signaling) and the following inhibitors: Y-27632 2HCl (ROCK inhibitor, 20 µM, Selleckchem), PD 98059 (MAPK-ERK inhibitor, 20 µM, Sigma-Aldrich, Saint Louis, MO, USA), SB 203580 (MAPK p38 inhibitor, 10 µM, Sigma-Aldrich), LY 294002 (PI3K inhibitor, Merckmillipore, 10 µM, Merck KGaA, Darmstadt, Germany), BAPTA-AM (Ca^2+^chelator, 10 µM, Enzo Life Sciences, Inc. Farmingdale, NY). No reduction in cell viability was observed in any treatment at the doses used.

### Small Interfering RNA (siRNA) Transfection

We purchased ON-TARGETplus SMARTpool siRNAs for WNT5A, FZD1, FZD2, FZD4, FZD5, FZD7, ROR1, ROR2, RYK, and control siRNAs from Dharmacon (Horizon Discovery Group, Cambridge, UK). RA FLS were cultured in six-well plates (15 x 10^4^ cells/well) and transiently transfected with 50 nM of siRNA in Opti-MEM I (Gibco, ThermoFisher, MA, USA) using DharmaFECT 1 (Dharmacon). The degree of suppression was determined by quantitative polymerase chain reaction (qPCR) or western blot.

### Proliferation Assay

RA FLS were cultured in 96-well plates (2 x 10^3^ cells/well) in DMEM, 5% FBS, 1% glutamine and 1% penicillin/streptomycin. Cells were treated with 400 ng/ml of rWnt5a for 24, 48, 72, and 96 h and proliferation was determined with the CellTiter-Glo luminescent viability assay (Promega, Wisconsin, USA) following the manufacturer’s instructions.

### Migration Assay

RA FLS migration was analyzed by a wound closure motility assay using Ibidi Culture Inserts (Ibidi, Martinsried, Germany) placed into a 24-well plate. FLS were seeded (15 x 10^3^ cells/well) in DMEM, 10% FBS, 1% Glutamine and 1% penicillin/streptomycin and treated with 400 ng/ml rWnt5a or other treatments when indicate. Microphotographs were taken 0 and 96 h after the treatment, and the areas of the remaining gaps were measured with the Image J software (National Institutes of Health, USA).

### Transwell Invasion Assay

The invasion assay was performed in 24-Well Milicell® Hanging Cell Culture Inserts that include a polyethylene terephthalate filter of 8.0 μm pore size (Merck Millipore, Darmstadt, Germany). The filter was coated with 200 µg/ml of the basement membrane Matrigel (BD Biosciences, Franklin Lakes, NJ, USA). On the upper chamber, a total of 5 × 10^4^ FLS were plated in 200 μl of DMEM, containing 1% FBS with or without 400 ng/ml rWnt5a. As chemoattractant, 750 μl of DMEM containing 10% FBS was placed on the lower chamber. The plates were then incubated for 48 h at 37°C in 5% CO_2_. Invading cells were fixed with paraformaldehyde and stained with Giemsa. Microphotographs of 10 random fields were taken in an Axio Vert.A1 (Zeiss, Oberkochen, Alemania) microscope at 20× magnification. The number of the cells was determined using the Image J software (National Institutes of Health, USA).

### Real-Time qPCR

Real-time qPCR was performed in duplicates using 1-Step QRTPCR-Brilliant III SYBR Green (Agilent Technologies, CA, USA) in a RotorGen (Corbett, Thermo Fisher Scientific) thermocycler, according to the manufacturer’s protocol. Relative levels of gene expression were normalized to the beta-actin gene using the comparative C_t_ method, where C_t_ is the cycle at which the amplification is initially detected. The relative amount of mRNA was calculated according to the 2^−ΔΔΔCt^ method, where: ΔC_t_ = C_t target_ − C_t Actin_ and ΔΔC_t_ = (C_t target_ − C_t Actin_)_Basal_ − (C_t target_ − C_t Actin_)_Treatment._ For the RA FLS treated as basal, ΔΔC_t_ = 0, and 2^0^ = 1. For the experimental treatments, the value 2^−ΔCt^ indicates gene expression relative to the beta-actin and the value 2^−ΔΔCt^ indicates the fold change in gene expression relative to the basal situation. The specific primers were from Qiagen (Hilden, Germany).

### Western Blot Analysis

After the indicated treatments, the proteins from the RA FLS were extracted using a cell lysis buffer. The protein concentration was determined with the Bradford assay (Bio-Rad Protein Assay; Bio-Rad, CA, USA). Protein samples (10–20 µg) were resolved in 8% gradient SDS-PAGE, transferred onto PVDF membranes (Merck Millipore, Darmstadt, Germany) and probed with primary antibodies directed against WNT5A (R&D), p44/42, SAPK/JNK, and p38 MAPK (MAPK Family antibody sampler kit, #9926), AKT (#9272), GSK3β(#9315), phospho-p44/42, phospho-SAPK/JNK, and phospho-p38 MAPK (Phospho-MAPK Family antibody sampler kit, #9910), phospho AKT (#9271), phospho GSK-3β (#9322), all from Cell Signaling Technology, Danvers, USA; and GAPDH antibody (Sigma-Aldrich). Bound antibodies were revealed with horseradish peroxidase-conjugated secondary antibodies (Cell Signaling and Santa Cruz Biotechnology), and blots were developed using the ECL Plus detection system (ChemiDoc™ MD Imaging System (Bio-Rad, California, EEUU)).

### Statistical Analysis

Differences between experimental groups were assessed with the Wilcoxon matched-pairs test or Mann Whitney U test. A value of p < 0.05 was considered significant. Analyses were performed with the GraphPad Prism software. (Prism 5.0 (GraphPad Software, San Diego, CA, EEUU).

## Results

### WNT5A Promotes Migration and Invasiveness of RA FLS

**Figure 1:**
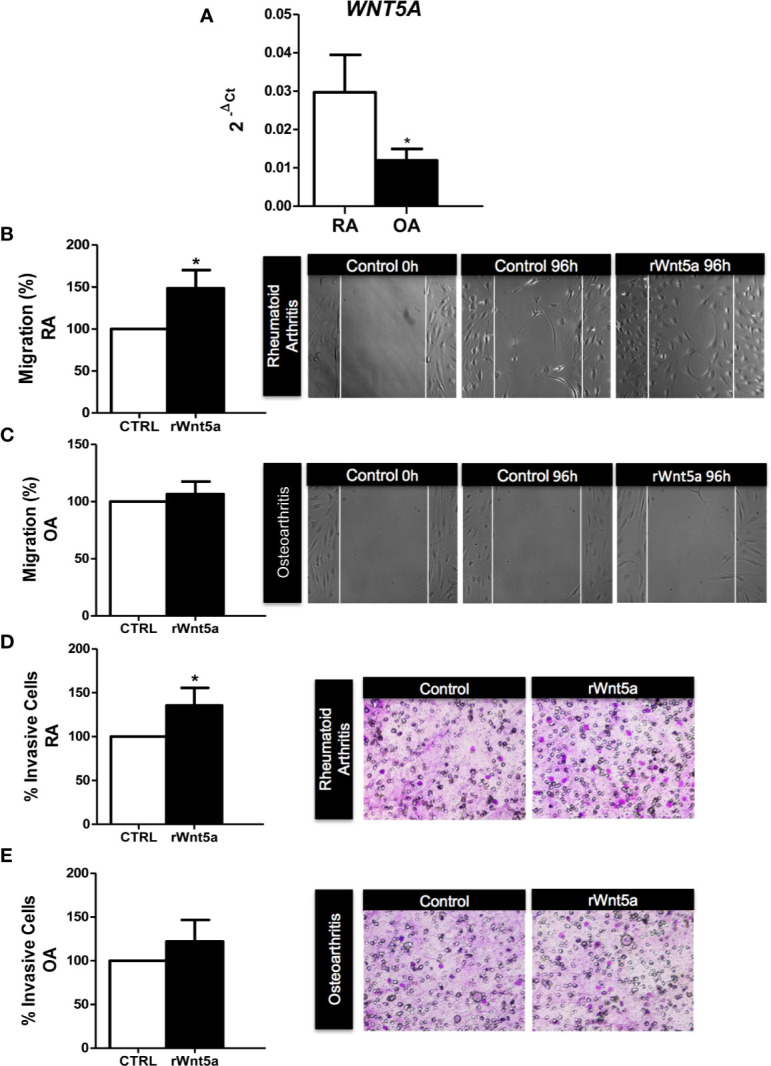
The migration and invasion of RA FLS are promoted by WNT5A. **(A)** Expression of *WNT5A* mRNA relative to that for β-actin in RA and OA patients was determined. **(B, C)** The FLS from RA **(B)** and OA **(C)** patients were stimulated with 400 ng/ml rWnt5a and the migration rate as percentage of the control was measured by wound-healing assays at 96 h **(D, E)** Percentage of RA FLS **(D)** and OA FLS **(E)** stimulated or not with 400 ng/ml rWnt5a, invading the Matrigel coated inserts at 48 h compared with the controls without rWnt5a. Values are the Mean ± Standard error of the mean (SEM) of FLS from 5 to 8 RA and OA patients obtained from a total of 9 and 8 independent experiments in migration and invasion assays, respectively. *P < 0.05, by Mann Whitney U test **(A)** and Wilcoxon matched-pairs test **(B–E)**.

We analyzed the mRNA expression of *WNT5A* in FLS from 7 RA and 5 OA patients by real-time PCR. As shown in **Figure 1A**, *WNT5A* mRNA was higher in RA FLS than in OA FLS (p<0.05). Next, we investigated the role of WNT5A on FLS migration using recombinant Wnt5a (rWnt5a) and a cell wound-healing assay. The preliminary experiments indicated that 400 ng/ml was the most effective rWnt5a dose (not shown), which is in accordance with previous studies (37, 38). FLS from 7 different RA patients and 5 OA patients were treated with 400 ng/ml rWnt5a for 96 h and the healed area was quantified. The migration of RA FLS treated with rWnt5a was significantly higher (48%) than without treatment (**Figure 1B**). However, this increase in migration was not observed in the OA FLS, as the healed area with OA FLS was similar with rWnt5a and without treatment (**Figure 1C**). Next, we investigated whether WNT5A could modify the invasiveness of the RA FLS. To this end, the transwell invasion assay through Matrigel was used. The results showed the RA FLS treated with rWnt5a were significantly more invasive (35% higher) than the non-treated RA FLS (**Figure 1D**). In contrast, addition of rWnt5a did not significantly increase the invasion ability of OA FLS (**Figure 1E**).

**Figure 2:**
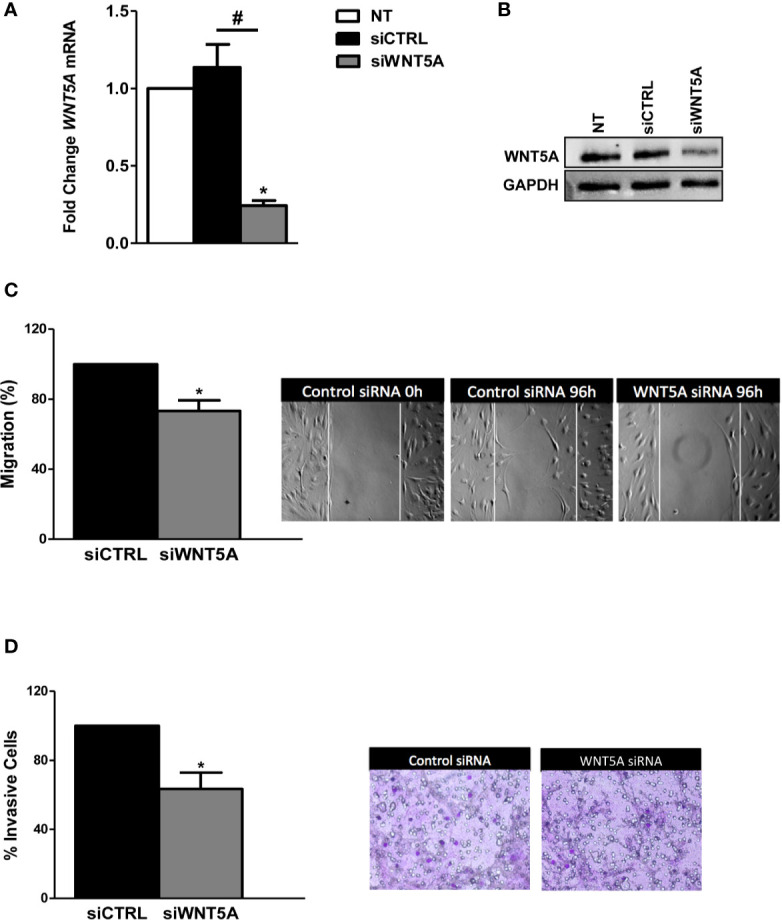
*WNT5A* suppression reduces migration and invasion in RA FLS. **(A, B)**. FLS were transfected with WNT5A or control siRNA and the efficiency of silencing was determined by real-time PCR **(A)** and western blot **(B)**. A representative blot is shown. **(C, D)** Analysis of migration **(C)** and invasion **(D)** in RA FLS transfected with WNT5A or control siRNA. Representative images are shown. Values are the Mean ± Standard error of the mean (SEM) of FLS from 6 patients obtained from 5 independent experiments in each assay. * and ^#^ indicates *P* < 0.05, by Wilcoxon matched-pairs test.

In the next experiments, we analyzed whether endogenous WNT5A modulates the migration and invasion of the RA FLS. These experiments were conducted after the knockdown of *WNT5A* by siRNA transfection, which reduced WNT5A expression by more than 75% at the RNA and protein levels (**Figures 2A, B**). The migration assay showed a significantly reduced healed area with the knocked FLS compared with the siControl transfected RA FLS (**Figure 2C**) that confirmed the migration-promoting role of WNT5A. Also, the RA FLS transfected with siWNT5A were significantly less invasive (37% lesser) than the siControl transfected FLS (**Figure 2D**).

Finally, we also analyzed the effect of WNT5A on the proliferation of the RA FLS. In this case, neither stimulation with rWnt5a nor silencing of the endogenous gene expression significantly modified the cells’ proliferation for up to 96 h compared with the controls (data not shown).

### WNT5A Promotes the Expression of Inflammatory Mediators and Metalloproteinases in RA FLS

**Figure 3:**
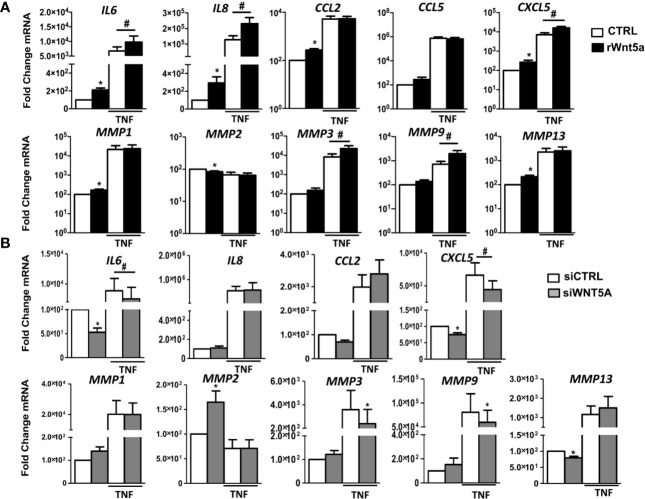
The gene expression of inflammatory mediators and metalloproteinases in RA FLS are promoted by WNT5A in RA FLS. **(A, B)** Fold change of *IL6, IL8*, *CCL2, CCL5, CXCL5, MMP1, MMP2, MMP3, MMP9*, and *MMP13* mRNA expression in RA FLS stimulated with 400 ng/ml rWnt5a alone or in combination with 10 ng/ml TNF **(A)**, or by transfection with *WNT5A* or control siRNA (*CCL5* was not included) **(B)**. Values are the Mean ± Standard error of the mean (SEM) of FLS from 7 patients with RA obtained from 7 independent experiments. * and # indicates P < 0.05, by Wilcoxon matched-pairs test.

We investigated the role of WNT5A in the spontaneous and TNF-induced inflammatory response of RA FLS. Then, we analyzed the mRNA expression of several chemokines, cytokines, and metalloproteases in the FLS from 7 RA patients. As shown in **Figure 3A**, rWnt5a induced the expression of IL6, IL8, CCL2, CXCL5, MMP1, and MMP13. The attained levels with rWnt5a were lower than those reached after TNF stimulation. However, the addition of rWnt5a to TNF further increased the expression of IL6, IL8, and CXCL5 over the induced by TNF alone (**Figure 3A**). In addition, rWnt5a potentiated the TNF induction of MMP3 and MMP9 by 2.3-fold and 2.7-fold, respectively, although it did not stimulate significantly their basal expression. The MMP2 metalloproteinase showed a contrasting pattern because it was significantly decreased, by rWnt5a (**Figure 3A**).

To further evaluate the effect of WNT5A on the inflammatory response of RA FLS, we analyzed whether suppression of endogenous *WNT5A* modulates the expression of inflammatory mediators and metalloproteinases that were induced by rWnt5a. As shown in **Figure 3B**, *WNT5A* suppression induced changes in expression that were almost completely opposite to the observed with the addition of rWnt5a. In detail, the basal mRNA expression of *IL6*, *CXCL5*, and *MMP13* was reduced, and the expression of *MMP2* was increased. In addition, *WNT5A* knockdown reduced the TNF-induced expression of *IL6*, *CXCL5*, *MMP3*, and *MMP9* (**Figure 3B**). Therefore, the two sets of experiments were widely concordant in their support of WNT5A as an inducer of inflammatory mediators.

### WNT5A Signaling in RA FLS Is Dependent on the RYK Receptor

**Figure 4:**
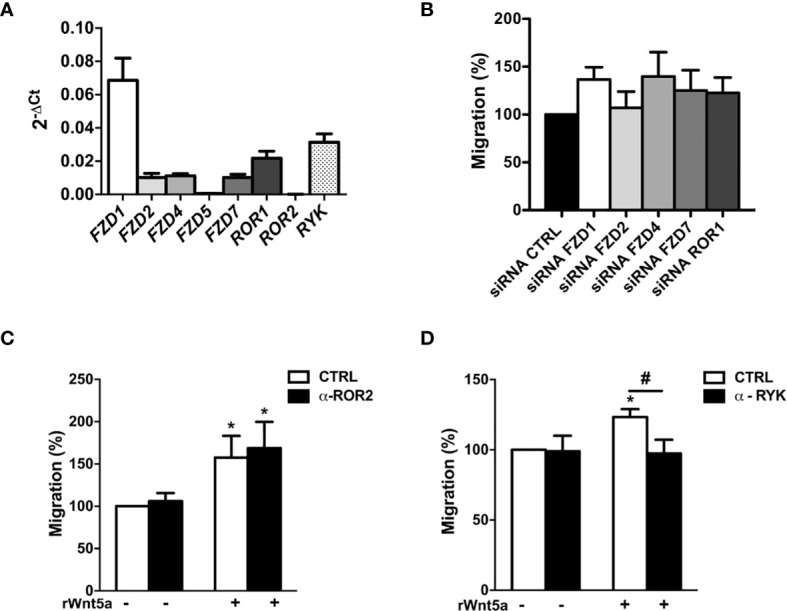
Identification of the receptors involved in WNT5A signaling in RA FLS. **(A)** Expression of the WNT receptors in FLS from 6 RA patients as assessed by real-time PCR. **(B)** Effect of silencing RA FLS *FZD1, FZD2, FZD4, FZD7* and ROR1 by siRNA transfection on the RA FLS migration stimulated with 400 ng/ml rWnt5a in comparison with the siRNA control transfection as measured by wound-healing assays at 96 h. **(C, D)** Effect of the anti ROR2 antibody (α-ROR2), 4 μg/ml **(C)**, or the anti-RYK antibody (α-RYK), 1 μg/ml **(D)** on the basal and rWnt5a-induced migration (400 ng/ml) of RA FLS. Migration of RA FLS without treatment was the 100% **(C, D)**. Values are the Mean ± Standard error of the mean (SEM) of FLS from 6 to 8 patients with RA obtained from four to seven independent experiments. * and # indicate P < 0.05, by Wilcoxon matched-pairs test.

To explore the mechanisms underlying the effects of WNT5A on RA FLS, we first analyzed the mRNA expression of the WNT5A receptors. The real-time PCR showed that six of the eight receptors were expressed at easily detectable levels. *FZD1* was the most highly expressed receptor followed by *RYK*, and at a lower level by *ROR1*, *FZD2, FZD4* and *FZD7* (**Figure 4A**). The ROR2 and FZD5 receptors were much lower. Then, we decided not to continue investigating FZD5 given the difficulties to achieve an adequate silencing due to its low expression and the absence of specific anti-FZD5 blocking antibodies or inhibitors. However, we considered worth pursuing ROR2 investigation given the reagent availability and that it has been previously involved in proliferation, migration and invasion of several cancer cells (39–41). Next, we analyzed the effect of the remaining receptors knockdown on the rWnt5a-induced migration (**Figure 4B**). We obtained efficient silencing of five of the seven genes as assessed by real-time PCR (data not shown). However, the knockdown of none of them, *FZD1*, *FZD2*, *FZD4*, *FZD7* and *ROR1*, modified the rWnt5a induction of FLS migration, as similar healed areas were observed in cells transfected with targeted siRNA and with siControl (**Figure 4B**). Therefore, our investigation proceeded with the two receptors without acceptable silencing, *ROR2* and *RYK*. They were targeted with blocking antibodies (42, 43). The anti-ROR2 antibody did not modify the basal or rWnt5a stimulated migration (**Figure 4C**). Only the treatment with anti-RYK antibody reverted the migration of FLS induced by rWnt5a (**Figure 4D**). Overall, these findings indicated that WNT5A promotes RA FLS migration by activating RYK-dependent non-canonical WNT signaling.

### WNT5A Stimulates RA FLS Migration via WNT/Ca^+2^ and RhoA/ROCK Pathways

**Figure 5:**
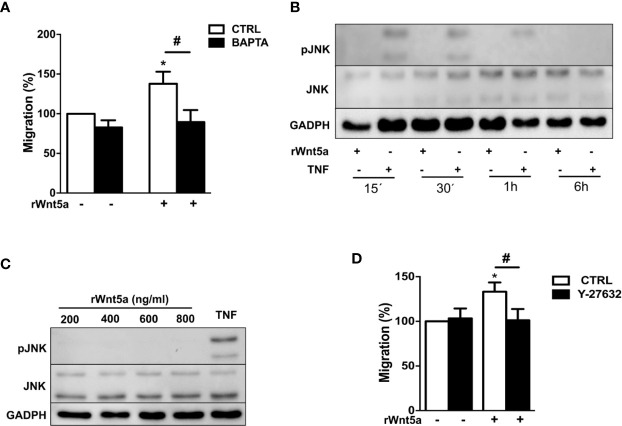
Identification of the pathways involved in WNT5A signaling in RA FLS. **(A)** Effect of the BAPTA Ca^2+^ chelator, 10 μM on the basal and rWnt5a-induced migration (400 ng/ml) of RA FLS. **(B)** Representative blot of the time-course analysis of the changes in JNK phosphorylation induced by incubation with 400 ng/ml rWnt5a in comparison with 10 ng/ml TNF as determined by western blot. **(C)** Representative blot of the activation status of the JNK MAPK in RA FLS treated with the indicated doses of rWnt5a for 1 h in comparison with 10 ng/ml TNF. **(D)** Impact of the Y-27632 ROCK inhibitor (20 μM) on the rWnt5a-induced migration of the RA FLS. Migration of RA FLS without treatment was the 100% **(A, C)**. Values are the Mean ± Standard error of the mean (SEM) of FLS from six to eight patients with RA obtained from 6 independent experiments. * and # indicate P < 0.05, by Wilcoxon matched-pairs test.

We examined which non-canonical WNT5A pathway, WNT/Ca^+2^ or WNT/PCP, is activated by WNT5A in the RA FLS. First, we addressed Ca^2+^ mobilization by treating the FLS from 7 RA patients with 10 μM of the selective calcium chelator 1,2-bis(2-aminophenoxy)ethane-N,N,N′,N′-tetraacetic acid (BAPTA). This chelator significantly reduced the rWnt5a stimulated FLS migration (**Figure 5A**). Therefore, the WNT/Ca^2+^ pathway may contribute to WNT5A-induced migration of RA FLS. Next, we analyzed the JNK activation after rWnt5a treatment. As shown in **Figure 5B**, JNK phosphorylation was not detected in the rWnt5a stimulated FLS, at any time from 15’ to 6 h, whereas it was observed at 15', 30' and 1 h after TNF stimulation. Also, rWnt5a did not induce JNK activation in RA FLS at any of the doses from 200 to 800 ng/ml for 1 h (**Figure 5C**).

Therefore, we analyzed the other branch of the PCP pathway by using the specific ROCK inhibitor, Y-27632. This inhibitor significantly reduced the migration rate of the rWnt5a stimulated FLS (**Figure 5D**). Overall these data indicated that WNT5A induces RA FLS migration by activation of WNT/Ca^+2^ pathway and the RhoA/ROCK branch of the WNT/PCP pathway.

### WNT5A Activates the p38, ERK MAPK, and PI3K/AKT Pathways in RA FLS Through the RYK Receptor and ROCK Signaling

**Figure 6:**
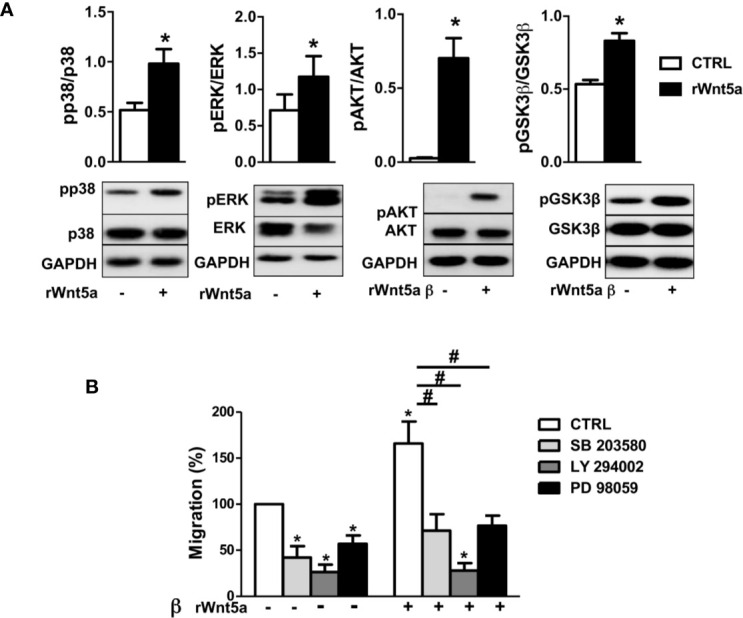
Downstream kinases activated by rWnt5a promote migration in RA FLS. **(A)** Analysis of the activation of p38, ERK MAPK, PI3K/AKT, and GSK3β by western blot in RA FLS treated with 400 ng/ml rWnt5a. **(B)** Effect of the MAPK-p38 inhibitor (SB 203580), PI3K inhibitor (LY 294002) or MAPK-ERK inhibitor (PD 98059) on the basal and rWnt5a-induced migration in RA FLS at 96 h. Values are the Mean ± Standard error of the mean (SEM) of FLS from six to eight patients with RA obtained from three to four independent experiments. * and # indicate P < 0.05, by Wilcoxon matched-pairs test.

The MAPK and PI3K/AKT signaling pathways have been previously involved in the migration of RA FLS (44, 45) and other cells (46). Therefore, we analyzed the effect of WNT5A on the activation of p38 and ERK MAPK, and of AKT and GSK3β of inhibiting the two pathways on RA FLS migration. The first part of the analysis consisted of the western-blot assessment of the kinases’ phosphorylation in the FLS from 6 RA patients after stimulation with rWnt5a for 1 h. The results showed evidence of the activation of the four analyzed kinases. Specifically, significantly higher phospho-p38, phospho-ERK, phospho-AKT and phosphor GSK3β were observed in the rWnt5a-stimulated FLS than in the FLS without treatment (**Figure 6A**). The second part of the analysis consisted of preincubating the RA FLS with specific inhibitors of the MAPK-p38 (SB 203580), MAPK-ERK (PD 98059) or PI3K (LY 294002) before measuring migration in the wound healing assay. The three inhibitors significantly reduced the migration of the RA FLS, both in basal conditions and after induction with rWnt5a, confirming the involvement of the two kinase pathways (**Figure 6B**).

**Figure 7:**
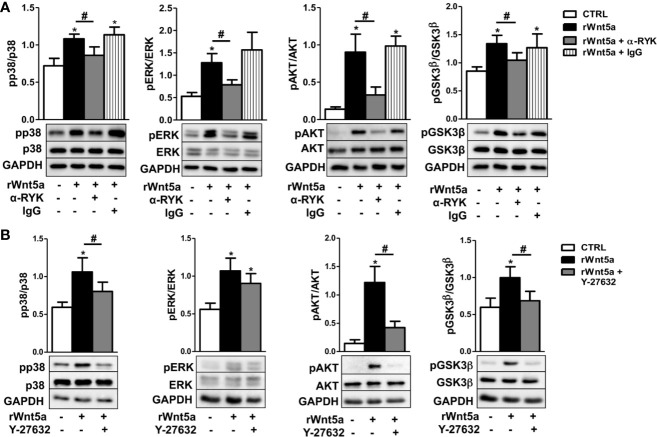
Contribution of RYK/ROCK pathway to the activation of p38, ERK MAPK, PI3K/AKT, and GSK3β kinases. **(A)** Representative western blot showing the effect of the anti-RYK antibody (α-RYK) on the rWnt5a-induced activation of p38, ERK MAPK, PI3K/AKT, and GSK3β in RA FLS. **(B)** Effect of the Y-27632 ROCK inhibitor (20 μM) on the rWnt5a-induced activation of p38, ERK MAPK, PI3K/AKT, and GSK3β kinases determined by Western blot in RA FLS. Values are the Mean ± Standard error of the mean (SEM) of FLS from six to eight patients with RA obtained from three to four independent experiments. * and # indicate P < 0.05, by Wilcoxon matched-pairs test.

Given that our results indicate WNT5A promotes RA FLS migration via the RYK receptor, we treated FLS from 6 RA patients with the anti-RYK antibody or IgG control and rWnt5a for 1 h. The western blot showed significant reductions of pP38, pERK, pAKT, and pGSK3β that were not observed with the IgG control (**Figure 7A**). In addition, we analyzed the effect of the ROCK inhibitor, Y-27632, given the dependence on ROCK activation of the rWnt5a-induced RA FLS migration. The experiments were conducted as with the anti-RYK antibody but replacing the antibody by the Y-27632 inhibitor. In this case, the Western blot showed a significant reduction of the phosphorylated p38, AKT, and GSK3β and a non-significant trend for diminution of the ERK activation (**Figure 7B**). These results are a confirmation of the involvement of the multiple pathways downstream of the RYK receptor that we have identified.

## Discussion

**Figure 8:**
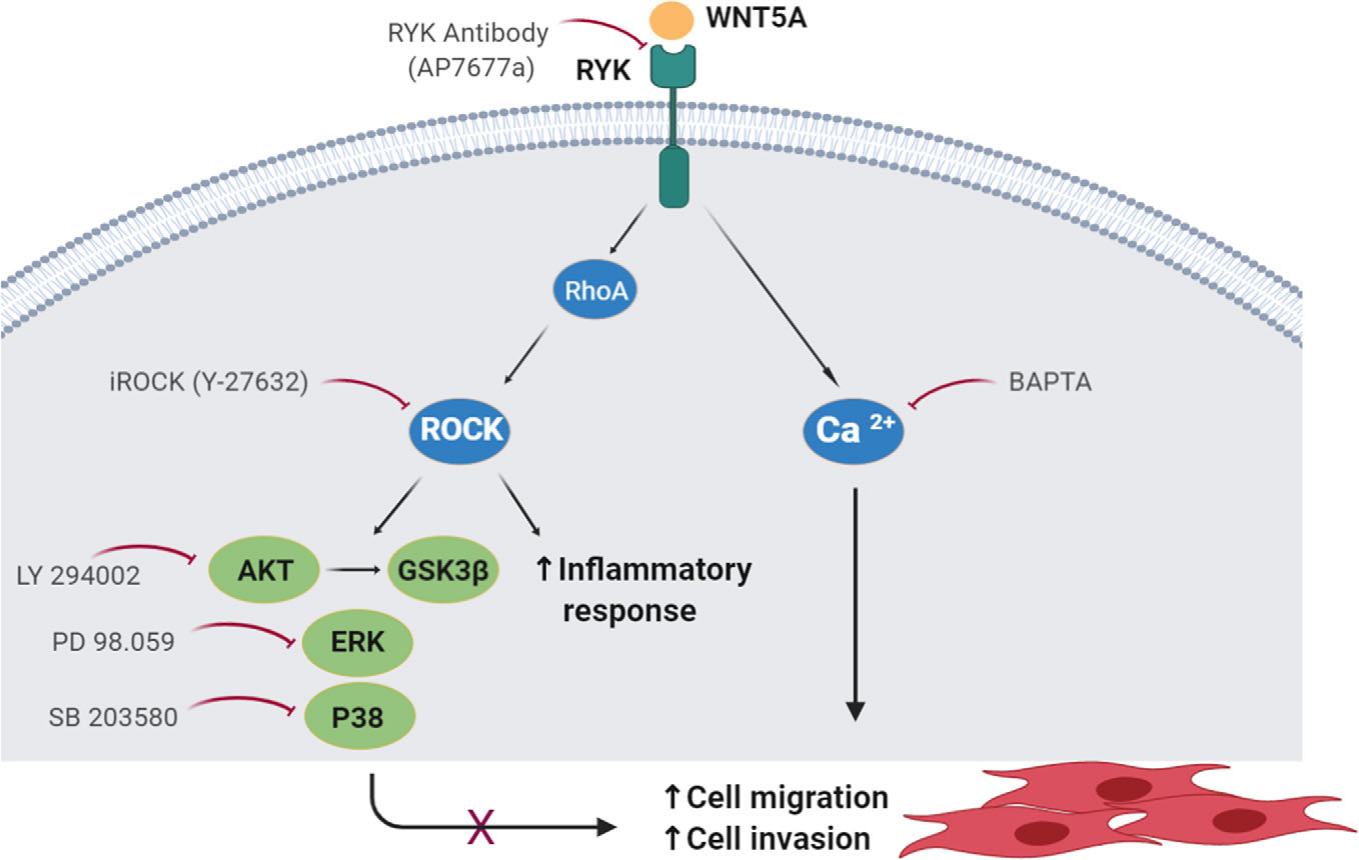
Schematic representation of the WNT5A signaling pathways involved in the migration of RA FLS. The participating molecules are shown together with the reagent used to demonstrate their respective involvement.

Our results indicate that WNT5A contributes to the aggressive phenotype of the FLS in RA patients because it promotes their enhanced migration and invasion, and the expression of inflammatory mediators via the WNT/Ca^2+^ and RYK/RhoA/ROCK signaling pathways (**Figure 8**). The regulation of RA FLS migration and invasion by WNT5A was demonstrated not only with the addition of rWnt5a, but most notably with the silencing of endogenous WNT5A expression. This modulation was specific of RA FLS since the migration and invasion of FLS from OA patients were not induced. This novel contribution of WNT5A to migration and invasion complements the already known participation of WNT5A to the induction of inflammatory mediators by these cells. In this respect, our study confirms the induction of IL6 and IL8, and shows that also CCL2, CXCL5, MMP1, MMP3, MMP9, and MMP13 are induced by rWnt5a in the RA FLS. Also, our results indicate that some mediators are more sensitive to WNT5A than others. The first group includes IL6 and CXCL5, which were modulated by WNT5A in RA FLS under the four analyzed conditions. In contrast, MMP13 was only responsive to WNT5A in the absence of TNF, whereas MMP3 and MMP9 were sensitive to WNT5A only in the presence of TNF, which could be a more relevant context for RA pathogenesis. Finally, IL8 and MMP1 were induced by rWnt5a, but they did not decrease after *WNT5A* silencing. This heterogeneity likely reflects the participation of a variety of pathways in the regulation of each of these mediators in the RA FLS. Our experiments also show that the promotion of migration requires Ca^2+^, RYK and ROCK, indicating the WNT5A signal could follow the WNT/Ca^2+^ and RYK/RhoA/ROCK signaling pathways. The latter led to the activation of p38, ERK MAPK and PI3K/AKT. In this way, the identification of WNT5A role is an unexplored aspect of the RA FLS phenotype, and delimitation of the implicated signaling pathway enriches our knowledge of RA pathogenesis and provides new targets for treatment.

Our results are consistent with the previously reported effect of WNT5A in embryonic development and several cancer types. In general, WNT5A regulates embryonic tissue morphogenesis through control of cell migration and polarization, rather than proliferation or fate speciﬁcation (13). This behavior is manifest in embryonic processes as elongation of the body axis, growth of long bones, digits, ribs and sternum, craniofacial morphogenesis, eyelid development and neuronal convergent extension and axon guidance (13, 15, 16). Regarding the involvement in cancer, overexpression of WNT5A significantly induced migration and invasion whereas WNT5A knockdown decreased them in cell lines from nasopharyngeal cancer (25). WNT5A also induced gastric cancer migration and invasion by binding to FZD2 and ROR2, and treatment *in vivo* with anti-WNT5A antibody inhibited liver metastasis of gastric cancer cells (47). In the same way, WNT5A induced migration in chronic lymphocytic leukemia cells (48) and melanomas (49). However, the role of WNT5A is complex and depending on other cell characteristics. This could be the reason explaining that opposite effects have been described in other cancer cells (50–52). This is a common feature of WNTs where a major determinant of the effects is the cellular context concerning factors such as the expressed repertoire of receptors and signal transducers (13). The lack of response to rWnt5a of the FLS from OA patients we have found represents another example of the importance of the cellular context. A context that differs in a broad range of epigenetic marks and gene expression patterns between RA and OA FLS (53, 54).

The cellular context could explain also the MMP2 expression changes in response to WNT5A. Only MMP2 among the explored inflammatory mediators was downregulated by rWnt5a (and upregulated by silencing *WNT5*A). This downregulation contrast with the decreased Mmp2 expression observed in Wnt5a cKO arthritic mice (33), although the two results are not fully comparable because of the complex mixture of tissues from the paw analyzed in the mouse. There are no other sources of information on the MMP2 regulation by WNT5A in arthritis, but in cancer cells the two types of results can be found. Several cells show induction of MMP2 by WNT5A (55, 56), but others show inhibition (57). The cancer studies are also of interest because they show that WNT5A stimulates MMP2 expression through ROR2 (55, 56), a receptor that was expressed at low levels in the RA FLS and that was not involved in the WNT5A modulation of migration suggesting a possible mechanism of the differential MMP2 regulation. The context-dependency is also a major determinant of the speciﬁc signaling pathway activated by the WNT ligand. The specific pathway activated by WNT5A in the RA FLS required the RYK receptor. RYK is an atypical receptor tyrosine kinase due to its inactive tyrosine kinase domain (58). It has been extensively studied in the development of nervous systems where it is important for establishing planar polarity during neural tube development and neuronal migration into the expanding cortical plate (16). However, it participates in many other systems as revealed in the Ryk loss-of-function mice with skeletal, craniofacial and cardiac abnormalities many of them overlapping with the observed in the Wnt5a null mice (58). This pathway of WNT5A migration promotion was in contrast with the followed by some other systems that require ROR2. Our results showed that ROR2 was both, expressed at very low levels and not required for migration of the RA FLS. These findings are of interest because ROR2 is known to recruit the actin binding protein filamin A leading to cytoskeleton remodeling and filopodial extension through JNK activation (59), which was also not observed in the RA FLS.

The two intracellular signaling pathways that were activated in our experiments were WNT/Ca^2+^ and the branch of the PCP pathway involving ROCK and the downstream p38/ERK/AKT/GSK3β kinases. The two are known to show a great deal of crosstalk and to cooperate in some processes leading to a significant overlap between their functions. As an example, the two overlap during development: PCP is important for cell migration, axon growth and pathfinding, synaptogenesis and ciliogenesis through modulation of the actin and microtubule cytoskeletons, and the WNT/Ca^2+^ pathway is important in cell fate determination, cell migration, embryonic convergent extension and axon guidance (16). Therefore, it is not surprising that the two pathways cooperate to potentiate FLS migration. The participation of ROCK is also very congruent with the best-known function of this member of the serine/threonine kinases, which is facilitating actomyosin cytoskeleton contractility. Specifically, ROCK is involved in the formation of stress fibers and focal adhesions (60).

The magnitude of the changes in migration and invasion was about a third of the baseline level. These changes are modest in comparison with the induced by some cytokines and chemokines (7, 61, 62), but similar to the observed in other studies (44, 63–65), including the study blocking the sonic hedgehog pathway (44) or suppressing cathepsin B (65). These examples and the reduced severity of arthritis in the Wnt5a cKO mice (33) support the relevance of the WNT5A modulation. In any case, the clinical benefit of inhibiting the WNT5A pathway will depend on its global contribution to RA, not only on the decreased migration and invasion. Indeed, blocking WNT5A signaling would have multiple therapeutic benefits, acting on the two aspects of RA pathogenesis, chronic inflammation and joint damage. Specifically, WNT5A is involved in the production of inflammatory mediators and metalloproteinases, in osteoclastogenesis and in FLS migration and invasion. Some of these benefits will be obtained by blocking downstream factors in the pathway. Accordingly, we showed here that treatment with the ROCK specific inhibitor, Y-27632, completely inhibited the migration of RA FLS induced by rWnt5a. In this regard, a previous study has shown that treatment with Fasudil, a less specific ROCK inhibitor approved for human use in Japan, suppressed cytokine expression in RA FLS and reduced the severity of arthritis in rats with adjuvant-induced arthritis (66).

A limitation of our study is the lack of distinction between ROCK1 and ROCK2. However, this is the commonest approach in current research because of the high degree of homology between the two, the many commonalities in their regulation, pattern of expression, and functions, and the fact that most inhibitors do not distinguish between them (including Y-27632 and Fasudil) (60). Another limitation is the impossibility to completely exclude signaling through FZD receptors due to the absence of specific reagents and the high degree of redundancy between them (67). Therefore, silencing the FZD one by one will not disclose their involvement, as happened with the individual FZD knockout mice that showed incomplete phenotypes due to signaling through alternative FZD (68). A circumstance that was only disclosed in the double knockout animals. This scenario should be considered because the role of RYK in some systems acts as a co-receptor associated with FZD receptors (16, 58, 67).

In summary, our discovery of the WNT5A/RYK/ROCK signaling pathway as a promoter of the enhanced migration and invasion of RA FLS and the definition of other components of the aggressive phenotype of the RA FLS has led to the identification of molecular targets that might be therapeutically beneficial in the RA patients.

## Data Availability Statement

The raw data supporting the conclusions of this article will be made available by the authors, without undue reservation.

## Ethics Statement

The studies involving human participants were reviewed and approved by Comité de Ética de Investigación de Santiago-Lugo (2017/521). The patients/participants provided their written informed consent to participate in this study.

## Author Contributions

AR-T performed the experiments and participated in the analysis of data and in drafting the manuscript. NM performed the experiments and participated in the analysis of data. CP performed the experiments. FR-T obtained the fibroblast-like synoviocytes from OA patients and participated in the experiments with these cells and in analysis of data. AM-V obtained the fibroblast-like synoviocytes from RA patients and participated in the analysis of data. AG participated in the analysis of data and wrote the manuscript. CC planed and managed the project, analyzed data and wrote the manuscript. All authors contributed to the article and approved the submitted version.

## Funding

This work was supported by Fondo de Investigación Sanitaria, Instituto de Salud Carlos III, with participation of European Regional Development Fund (FEDER) funds (European Union) [Grant PI1701660 and by Redes Temáticas de Investigación Cooperativa en Salud (RETICS) Program, RD16/0012/0014].

## Conflict of Interest

The authors declare that the research was conducted in the absence of any commercial or financial relationships that could be construed as a potential conflict of interest.
